# Subcutaneous Lecanemab: Potential Benefits and Place in Therapy

**DOI:** 10.1002/alz70859_104695

**Published:** 2025-12-25

**Authors:** Sharon Cohen, Erica Andreozzi, Steven Hersch, Michelle Gee, Michael C. Irizarry, Lynn D Kramer

**Affiliations:** ^1^ Toronto Memory Program, Toronto, ON Canada; ^2^ Eisai Inc., Nutley, NJ USA; ^3^ Eisai Ltd., Hatfield United Kingdom

## Abstract

**Background:**

Lecanemab is a novel, humanized monoclonal antibody, which has demonstrated the ability to substantially reduce markers of amyloid and slow clinical decline on multiple measures of cognition and function in early Alzheimer’s disease (AD). A subcutaneous administration of lecanemab is in development, to improve patient and caregiver convenience relative to the intravenous route of administration as well as eliminate the time and resources needed for preparation and administration of intravenous infusions. Herein, we highlight the potential benefits and place in therapy for a subcutaneous formulation of lecanemab based on currently available data and relevant literature.

**Methods:**

Utilizing the currently available data, expert consensus treatment guidelines, and available AD literature, we will place the results of the subcutaneous lecanemab clinical program in context of the current treatment paradigm in early AD. Results of recent human factors validation study which evaluated whether the new lecanemab autoinjector can be safely and effectively used under the expected use environments will also be presented. The human factors study assessed task performance of 110 trained and untrained participants (patients with self‐ or caregiver‐reported diagnosis of mild cognitive impairment or mild AD [n=63], caregivers [n=32], and healthcare providers [HCPs; n=15]) using the lecanemab autoinjector under the expected use environments/conditions without patterns of use error which could cause serious harm to a user, where harm is defined to include compromised medical care (e.g., incomplete dosing).

**Results:**

The continuous nature of AD pathophysiological process requires ongoing therapy. Given the burden of frequent intravenous injections, a subcutaneous lecanemab formulation would be a welcome addition for providing on‐going maintenance therapy. The newly developed lecanemab autoinjector offers an additional administration option with a weekly dosing schedule while enabling easier administration, increased access to the medication, and reduced burden. The human factors study revealed that a majority of HCPs (80%; 12/15), caregivers (84.4%; 27/32), and patients (82.5%; 52/63) were observed to administer a full dose successfully.

**Conclusions:**

Weekly subcutaneous lecanemab has the potential for a prominent place in therapy by providing comparable efficacy relative to intravenous formulation with a more convenient and accessible administration by patients, caregivers or HCPs.

